# Resistance to Neoadjuvant Treatment in Breast Cancer: Clinicopathological and Molecular Predictors

**DOI:** 10.3390/cancers12082012

**Published:** 2020-07-22

**Authors:** María Rosario Chica-Parrado, Ana Godoy-Ortiz, Begoña Jiménez, Nuria Ribelles, Isabel Barragan, Emilio Alba

**Affiliations:** 1 Institute of Biomedical Research in Malaga (IBIMA), Regional and Virgen de la Victoria University Hospitals Campus de Teatinos s/n, 29010 Málaga, Spain; ana.godoy.sspa@juntadeandalucia.es (A.G.-O.); bego.jimenez@ibima.eu (B.J.); nuria.ribelles.sspa@juntadeandalucia.es (N.R.); ealbac@uma.es (E.A.); 2 Cancer Molecular Biology Group, Medical Research Center, University of Málaga (UMA), C/Marqués de Beccaría n°3, 29010 Málaga, Spain; 3 Medical Oncology Service Intercentros, Regional and Virgen de la Victoria University Hospitals, IBIMA, Campus de Teatinos s/n, 29010 Málaga, Spain; 4 Group of Pharmacoepigenetics, Department of Physiology and Pharmacology, Karolinska Institutet, 17177 Stockholm, Sweden

**Keywords:** breast cancer, neoadjuvant chemotherapy, pathological complete response, predictive markers, residual disease

## Abstract

Neoadjuvant Chemotherapy (NAC) in Breast Cancer (BC) has proved useful for the reduction in tumor burden prior to surgery, allowing for a more extensive breast preservation and the eradication of subjacent micrometastases. However, the impact on prognosis is highly dependent on the establishment of Pathological Complete Response (pCR), in particular for Triple Negative (TN) and Hormonal Receptor negative/Human Epidermal growth factor Receptor 2 positive (HR−/HER2+) subtypes. Several pCR predictors, such as PAM50, Integrative Cluster (IntClust), mutations in PI3KCA, or the Trastuzumab Risk model (TRAR), are useful molecular tools for estimating response to treatment and are prognostic. Major evolution events during BC NAC that feature the Residual Disease (RD) are the loss of HR and HER2, which are prognostic of bad outcome, and stemness and immune depletion-related gene expression aberrations. This dynamic nature of the determinants of response to BC NAC, together with the extensive heterogeneity of BC, raises the need to discern the individual and subtype-specific determinants of resistance. Moreover, refining the current approaches for a comprehensive monitoring of tumor evolution during treatment, RD, and eventual recurrences is essential for identifying new actionable alterations and the integral best management of the disease.

## 1. Neoadjuvant Chemotherapy in Breast Cancer Treatments

Neoadjuvant Chemotherapy (NAC) is widely used as a standard of care for early and locally advanced Breast Cancer (BC). The standard practice consists of the administration of anthracycline-based chemotherapy and subsequent treatment with a taxane, with the addition of anti-Human Epidermal Growth Factor Receptor 2 (HER2) therapy in HER2+ disease [1]. The main purposes of NAC in the clinical setting are the increase in breast preservation rate (reduction in tumor burden) and the achievement of a pathological Complete Response (pCR), which greatly influences Disease-Free Survival (DFS) and Overall Survival (OS). In addition, the implementation of NAC became a driving force in the search for new therapeutic targets and generated excellent in vivo models to investigate the sensitivity and resistance mechanisms of novel therapeutic approaches.

The response to NAC is key for the assessment of outcome after surgery, which is based on the pathological examination of breast tissue and lymph nodes that are surgically removed after NAC [2,3]. This assessment can be dichotomized in pCR and Residual Disease (RD). pCR is defined as the absence of invasive cancer cells in the breast and/or axillary nodes (ypT0/Tis ypN0), while RD is defined as the non-absence of invasive cancer cells in the breast and axillary nodes. Within this frame, several types of grading and classifications of pCR and RD have been established depending on the employed pathological examination (reviewed in Penault-Llorca et al., 2016 [3]). Classifications such as Miller and Payne [4] and Residual Cancer Burden (RCB) [5] quantify the response to NAC in several grades, where one is pCR and the others correspond to a spectrum of values that reflect the extent of RD [3].

The association of sensitivity to NAC in terms of pCR achievement with better long-term outcomes has been established [6], particularly in specific BC subtypes, such as luminal B/HER2 negative (HER2−), HER2-positive (HER2+)/non-luminal, and Triple Negative (TN) BC [7].

Different studies from Collaborative Trials in Neoadjuvant Breast Cancer (CTNeoBC) showed that the absence of invasive tumor cells in breast and axillary lymph nodes after NAC added prognosis value when using Event-Free Survival (EFS) and OS as outcome measurements, to the sole detection of breast invasive tumor cells (EFS HR 0.44 vs. 0.6; OS HR 0.36 vs. 0.51) [8]. In general, patients that have pCR following NAC are much less likely to recur than those with RD [6,9,10,11,12]. The approximate general percentage of pCR in BC is 31%, whereas pCR achievement in BC subtypes is 12% for HR+/HER2−, 40% for TN, and 47% for HER2+ (anti-HER2 treated) subtypes [12]. The subtype-specific associations between pCR and outcome arose in three pivotal clinical trials, NeoALTTO [13], NOAH [14], and NeoSphere [15], which collectively show that pCR is a highly informative prognosis biomarker in the HER2+ subtype; those patients with HER2+ breast tumors treated with NAC and anti-HER2 treatment (Trastuzumab or Trastuzumab plus Pertuzumab) that achieved pCR had a significantly higher 6-year EFS (77% vs. 65%) and OS (86% vs. 77%) compared with those without pCR [16]. The association between pCR and long-term outcomes in the different BC subtypes has been reviewed elsewhere [6]; when pCR is documented, the risk of death was reduced by 84% in TN, 92% in HER2+, and 71% in HR+ BC. The most relevant recent clinical trials about NAC treatment with pCR as endpoint are described in Table 1. Specific recent reviews on clinical trials evaluating NAC in TN and HER2+ BC give a good account of them [17,18].

Given this relevant potential as a prognostic biomarker, and considering the discrete percentage of patients that achieve pCR after NAC (31%) [12], there is an urgent need to define those predictive factors that can anticipate response to NAC, and therefore, cooperate to determine the best therapeutic approach in advance.

## 2. Predictive Factors of Pathological Complete Response

The utility of pCR as a surrogate of outcome has raised growing interest in identifying those biomarkers that predict pCR, particularly in a scenario where NAC fails in more than 70% of patients [12].

Therefore, defining the biomarkers that can discern between responder and non-responder patients before NAC could help to determine the initial pharmacological treatment or to decide whether to apply breast conservative surgery at the start of the therapeutic approach.

### 2.1. Clinicopathological Factors

#### 2.1.1. cT-Stage

The tumor size, expressed as cT-stage, has been identified in several studies as an independent predictor of pCR. Prat et al. (2015) studied a cohort of 957 BC (10.3% HER2-enriched, 18.2% Luminal B, 30.6% Luminal A, and 32.7% Basal-like by PAM50 classification) treated with NAC, and showed that the most significant variable associated with pCR was cT-stage (*p* < 0.001). Half of the patients with cT3-4 reached pCR compared to patients with cT1-2 (12% vs. 24%) [28]. Additionally, in a cohort of 2046 patients evaluated by Goorts et al. (2017), of all of the tested variables (Estrogen Receptor (ER) status, Progesterone Receptor (PR) status, HER2 amplification, and tumor grade), the most important predictor of pCR was cT-stage [9]. For cT1 patients, pCR was achieved in 31%, 22.4% of those with cT2 showed pCR, and response success rates were 17.6% and 16.5% in cT3 and cT4, respectively. Lower cT-stage (cT1-2 vs. cT3-4) was a significant independent predictor of pCR (*p* = 0.001, OR 3.15). In the same study, positive HER2 status, negative ER, and negative PR were also pCR predictors [9].

#### 2.1.2. Tumor Grade

Another pathological factor associated with pCR after NAC is tumor grade. Different studies have shown that histological grade can predict the response to treatment [29,30]. In 2013, Lips et al. studied in a cohort of 560 primary BC (51.3% ER+, 11.6% ER+/HER2+, 10% HER2+, and 27.1% TN) tumors treated with NAC and determined that a high histological grade (G3) was the best predictor of pCR in ER+/HER2− tumors (*p* < 0.004) [29]. Interestingly, in TN patients, this association could be further refined according to specific NAC treatments, where G3 only predicted pCR in the arm of the cohort receiving platinum-based therapy (*n* = 121) (OR 2.27) [30]. Moreover, in a cohort of 353 patients with BC treated with NAC (55.8% HR+, 15.6% HR+/HER2+, 9.6% HER2+, and 19% TN), the multivariate analysis with other clinicopathological factors, such as Ki67, ER, PR, HER2, and negative status for ER, PR, and HER2 (TN), yielded tumor grade and ER status as independent predictors of pCR (*p* < 0.004). Tumor grade 3 versus the other grades was associated with pCR (OR 6.36, *p* = 0.004). In univariate analysis, tumor grade was noticeably associated with pCR status in patients with G1 and G2 tumors, where pCR rarely occurs, while 31.3% of G3 tumors had pCR (*p* < 0.0001) [31]. Finally, in 2019, Diaz-Redondo et al., in a HER2+ cohort of 259 patients treated with NAC-Trastuzumab ± Pertuzumab, determined that histological grade 3 was significantly related to pCR in a multivariate analysis [32].

#### 2.1.3. Immunohistochemical Subtypes

Irrespective of the type of predictor of pCR, it is important to emphasize that the survival advantage associated with the achievement of pCR is limited to specific breast cancer subtypes [8], as stated before, and is probably related to the pCR achievement bias that is inherent to BC-subtype. In locally advanced breast cancer (LABC), multivariate analysis indicated that 48% of patients with the overexpression of HER2 (treated with NAC plus Trastuzumab ± Pertuzumab) achieved pCR versus 7% of patients with the HR+/HER2− subtype, or 23% of TN patients; both of the latter groups were only treated with NAC (*p* < 0.001) [13]. In young women with breast cancer, the pattern of pCR achievement was also subtype-specific, with such an achievement being more frequent in the HER2+ subtype (47%) [12], as shown in another study that evaluated 6337 patients with primary breast cancer treated with NAC from seven randomized trials [7]. Apart from HER2 status, hormone receptor status was also found to be one of the best predictors of pCR, both by univariate and multivariate analysis in the study by Gentile et al. in 2017, with 321 LABC patients distributed as follows: 43% HR+/HER2−, 23% TN, and 34% HER2+ subtypes. The overall pCR rate was 25%, whereas those patients with HR+/HER2− subtypes achieved a rate of 7%, those with TN subtype 23%, and those with HER2+ tumors achieved a pCR rate of 48% (*p* < 0.001) [33]. Indeed, the fact that the pCR rate is so low for most HR+ breast cancers that chemotherapy does not seem to be useful in these cases constitutes a good example of the use of predictors of response to NAC for treatment decision-making algorithms [34].

#### 2.1.4. Pre-Treatment Ki67

Among the clinicopathological predictive factors, pre-therapy Ki67 has emerged as a promising candidate [3]. The immunohistochemistry (IHC)-based quantification of Ki67 in pre-treatment core biopsies is predictive of pCR, consistent with the anti-mitotic major mechanism of action of NAC. In a study of 262 patients, the proportion of patients with breast cancer achieving pCR was significantly higher among those presenting Ki67 >50% than among patients with Ki67 < 50% (40% and 19%, respectively; *p* = 0.0004). ER−/HER2+ was the subtype associated with the highest pCR rate (49%) and was also in patients with tumors with pre-therapy Ki67 >50% (63.6%), followed by patients with TN (41.7%) [35]. In a TN cohort, the pCR rate was 3.36-times higher in patients with high Ki67 expression than those with low expression [36]. Similar results were found in a recent study (2020) by Gluz et al. in a cohort of TN (*n* = 642 patients divided in two arms: 306 treated with nab-Paclitaxel + Carboplatin and 336 treated with nab-Paclitaxel + Gemcitabine), where the main objective was to find a predictive marker of pCR in non-anthracycline NAC; in both treatment arms, high Ki67 was a strong predictor of a high ratio of pCR (*p* < 0.001) [20]. The fact that this correlation has been observed primarily in TN cohorts is in good agreement with the well-known proliferative TN nature. Indeed, in ER+ BC, which is not enriched in cell proliferation pathways, the pre-treatment Ki67 index did not show any predictive value regarding pCR achievement [3,37].

#### 2.1.5. Tumor-Associated Lymphocytes

Based on previous evidence of the contribution of the immune system in chemotherapy response, in 2010, Denkert et al. evaluated the presence of tumor-associated lymphocytes as a predictor of pCR by IHC detection of CD3, CD20, and CXCR3 in a training cohort from the GeparDuo trial (*n* = 218), and a validation cohort from the GeparTrio cohort (*n* = 840). In both cohorts, the percentage of intratumor lymphocytes was a significant independent predictor of pCR (*p* = 0.012 and *p* = 0.001, respectively) [38]. They further validated this association in a cohort of 3771 patients, 1366 Luminal-HER2−, 1379 HER2+, and 906 TN, from six randomized trials organized by the German Breast Cancer Group [39]. They identified the following tumor infiltrating lymphocytes (TILs) thresholds for an association with pCR in all BC subtypes (*p* < 0.0001): low (0–10%), intermediate (11–59%) and high TILs (>59%). According to their results, TN is the BC subtype with the higher chance of benefiting from the presence of TILs (31% vs. 59% of pCR). In view of the proven BC immunogenicity and the benefit of immune influence on the response to NAC, they suggest that BC might be targetable by immune-modulating therapies [39]. In line with this, in a recent study (2019), Cerbelli et al. showed the potential of CD37 expression and TILs to predict response to NAC in a TN cohort (*n* = 61). Both the presence of TILs and CD37 expression were associated with a better pCR rate (*p* = 0.037, univariate; *p* = 0.011 and *p* = 0.014, univariate and multivariate, respectively) [40]. Taking into consideration not only these associations of tumor microenvironment-specific features with pCR, but also the groundbreaking therapies based on reactivation of tumor immune suppression in the exhausted inflamed tumor scenario, we anticipate that tumor-associated lymphocytes will be key for the assessment of response to neoadjuvant therapeutic approaches.

### 2.2. Molecular Determinants of pCR

While pCR has been extensively interrogated for its association with improved treatment outcome in patients treated with NAC and surgery, there are only a few studies that have explored the molecular determinants of pCR. In them, some predictive molecular factors of pCR have been reported, of which the most relevant are summarized in this section.

#### 2.2.1. PAM50 Intrinsic Subtypes

Among the pCR predictors that are currently in use, PAM50-defined molecular subtypes stand out as valuable tools to stratify patients based on expected pCR achievement. The refined molecular stratification that intrinsic subtypes provide incorporates the tumor biological scenario that can be associated with the NAC response.

The PAM50 gene expression signature establishes four different intrinsic subtypes: luminal A, luminal B, HER2-enriched, and basal-like. Importantly, intrinsic subtype was outlined as the only significant predictor of pCR against the different clinicopathological parameters, both in univariate (*p* = 0.007) and multivariate analyses (*p* = 0.031), defining those patient subgroups that are most likely to benefit from an adjuvant chemotherapy regimen [34].

Studies evaluating the predictive capacity of PAM50 subtypes have proliferated over the last decade, confirming its prominent position among the pCR predictors (Table 2).

The added value of PAM50 subtyping in the prediction of pCR is most relevant for the discrimination of those HER2+ tumors that are susceptible to benefit from NAC. In 2014, in a continuation of the NOAH study, with 114 HER2+ BC patients treated with NAC-cyclophosphamide/methotrexate/fluorouracil (CMF)+Trastuzumab, Prat et al. (2014) determined that patients with the HER2-enriched intrinsic subtype presented a comparatively higher ratio of pCR (Odds Ratio (OR) = 5.11, *p* < 0.009) [41]. This was also determined in another study that included the four IHC subtypes (*n* = 957), distributed as follows: 53.4% ER+, 46.6% PR+, 10% HER2+, and 36.6% TN. Here, the intrinsic subtype classification showed that, again, HER2-enriched subtype was associated with a high pCR rate (37%), as well as basal-like (38%), and, to a lower extent, luminal B subtype (16%) (*p* < 0.001, *p* < 0.001, and *p* < 0.001, respectively) [28].

Consistent with this and reinforcing the specificity of the HER2-enriched subtype for pCR prediction even among HER2+ tumors, in 2016, Dieci et al. identified the HER2-enriched subtype as the one with the highest rate of pCR, as opposed to luminal A, which showed the lower pCR rate (*p* < 0.026). The selected HER2+ patients (*n* = 121) were treated with NAC + Trastuzumab, NAC + Lapatinib or NAC, and presented the following distribution of intrinsic subtypes: 26.7% HER2-enriched, 25.6% luminal A, 16.3% luminal B, 14% basal-like, and 17.4% normal-like [25]. Another study from 2015 where the PAM50 predictive power has been tested in a HER2+ cohort, treated in NAC with Paclitaxel-Trastuzumab ± Lapatinib without anthracyclines, was undertaken by Carey et al. In 305 patients, the pCR rate was different across the intrinsic subtypes. Luminal A showed the lowest ratio (34%) and HER2-enriched achieved the highest (70%). However, no changes were observed between dual and single therapy [23]. Interestingly, the HER2-enriched subtype and subsequent treatment with HER2 blockade therapy (Lapatinib and Trastuzumab) but not with NAC, has been also associated with a higher pCR rate than the non-HER2-enriched subtypes: 41% of HER2-enriched patients achieved pCR versus 10% of non-HER2-enriched patients (OR 6.2, *p* = 0.0004) [44]. In the BERENICE trial, the response to Pertuzumab, Trastuzumab, and common neoadjuvant anthracycline-taxane regimens for HER2+ BC tumors was evaluated; the highest pCR rate was achieved again in HER2-enriched PAM50 subtype tumors, further consolidating the notion of the increased clinical benefit of the addition of Pertuzumab to NAC + Trastuzumab in HER2+ BC treatment [27].

Finally, in a recent study, Diaz-Redondo et al. evaluated the PAM50 signature as a predictor of pCR in a HER2+ cohort treated with NAC-Trastuzumab (Group A), or with NAC-Trastuzumab + Pertuzumab (B) cohort (*n* = 259). Group A (NAC-Trastuzumab) included more tumors of the HER2-enriched subtype, and 39% of the patients achieved pCR; on the other hand, Group B (NAC-Trastuzumab + Pertuzumab) was enriched in basal-like tumors, and had a pCR rate of 61%. The luminal subtypes were similarly distributed in both groups. In terms of pCR predictive potential of PAM50-defined molecular subtypes, in both cohorts, and consistent with the previously mentioned studies, the intrinsic subtype was correlated with pCR (*p* < 0.0001), independent of treatment, with HER2-enriched and basal-like being the most pCR favorable subtypes, and luminal A the subtype associated with the lowest pCR rate. Interestingly, the classic classification of BC by IHC had a lower predictive potential than intrinsic subtypes (IHC-luminal subtype, *p* = 0.03 and IHQ-HER2+ subtype, *p* = 0.06). Again, the clinical benefit of dual anti-HER2 therapy is exemplified by the differences in pCR achievement between Group A and B when the intrinsic subtype is considered; whereas luminal PAM50 subtypes and the HER2-enriched subtype present higher pCR rates in Cohort B (41% vs. 11.4%, *p* < 0.01, and 73.5% vs. 50%, *p* < 0.01, respectively), the rate of pCR is not significantly different in basal-like subtypes (53.3% vs. 50%), consistent with the absence of HER2 targets in basal-like tumors [32].

Apart from discriminating pCR-relevant subtypes within HER2+ BC, PAM50 signature is also useful for separating potential responders to NAC among TN and ER+ patients. In a cohort of TN patients (*n* = 1055) [42], this population was stratified according to the intrinsic subtypes, with basal-like being the most frequent (55–81%, depending on the dataset). Within this subtype (*n* = 56), both the high expression of the proliferation score or the low expression of the luminal A signature showed significant associations with pCR (*p* < 0.005 and *p* < 0.023, respectively) [42]. Another study, derived from the WSG-ADAPT-TN trial, was designed to identify predictive markers of pCR in a TN cohort treated with different combinations, such as nab-Paclitaxel + Carboplatin or nab-Paclitaxel + Gemcitabine; basal-like PAM50 was independently associated with pCR (*p* < 0.015, 38% basal-like vs. 20% non-basal-like) [20].

Regarding ER+ tumors, in 2016, Prat et al. showed that the luminal A subtype again predicts a low rate of pCR in an ER+ cohort (*n* = 180) in comparison with the other subtypes (*p* < 0.037) [43]. Moreover, a recent study based on ER+ tumors showed that luminal A is the intrinsic subtype associated with the lowest pCR rate [34].

Based on these data, PAM50 subtypes are positioned as a useful tool for evaluating by pCR achievement prediction, the response to NAC, and in the case of HER2+ BC, the response to NAC+anti-HER2 target therapies, with the general consensus of HER2-enriched and basal-like subtypes predicting higher pCR rates, and luminal A tumors predicting the lowest rates (Table 2). Of note, intrinsic subtypes can further discriminate the good responders to anti-HER2 therapies within HER2+patients.

#### 2.2.2. Other Genomic, Transcriptomic, and Proteomic Signatures

State of the art analyses are starting to unveil the contribution of single gene genomic and expression variants as candidate predictors of pCR. There have been many attempts to identify a gene or molecular signature that could predict pCR, which are summarized in Table 3.

##### Single Gene Variants as Predictors of Pathological Complete Response

The majority of studies describe the association of molecular variations of key cancer genes with pCR, such as *MYC* amplification in a cohort of 51 patients distributed in luminal A (22), luminal B (14), HER2+ (8), and TN (7) BC subtypes, all of them treated with neoadjuvant anthracycline-cyclophosphamide followed by taxanes. The *MYC* proto-oncogene amplification, defined as a ratio of MYC signals to chromosome 8 signals (MYC/CEP 8) >2.2, was associated with pCR in univariate logistic regression (*p* = 0.003). Moreover, the ROC curve assessing the sensitivity and specificity of the correlation of *MYC* copy number and pCR, shows that the MYC/CEP 8 ratio discriminates patients with pCR from those without pCR, with an area under the curve (AUC) of 87.8% (*p* = 0.006); this implies the high pCR predictive potential of *MYC* amplification in the context of treatment with AC+T in NAC [46]. Another new relevant gene in cancer, *SPAG5*, is reported as a transcript (in the MD Anderson-NeoACT trial cohort; *n* = 508), a protein (in the Nottingham-NeoACT trial cohort; *n* = 200), and as an independent predictor of pCR in multivariate logistic regression (*p* < 0.024 and *p* < 0.0010) [45].

A study derived from the GeparSepto trial, which screened a hotspot panel in 16 commonly mutated genes in all breast cancer subtypes and Copy Number Alterations (CNAs) in 8 cancer-relevant genes, concluded that the genes harboring the majority of genetic variants among the different subtypes were *TP53* and *PIK3CA*. On the other hand, genetic heterogeneity in different breast cancer subtypes (Luminal/HER2− vs. HER2+ vs. TN) were related to differences in NAC response; *TOP2A* amplification was associated with a decrease in pCR in the TN cohort (multivariate *p* < 0.006). Mutated *PIK3CA* was associated with a reduced pCR (*PIK3CA*mut: 23% vs. *PIK3CA*wild-type 38.8%, *p* < 0.0001), particularly in the HER2+ cohort (multivariate OR = 0.43, *p* < 0.006) [49]. Consistent with these findings, in a HER2+ cohort from the NeoSphere trial, it was observed that the mutation in exon 9 of *PIK3CA* produced a lack of sensitivity to anti-HER2 therapies [47]. Hence, the mutated status of *PIK3CA* could be a prognostic factor of treatment resistance with NAC and anti-HER2 therapies. This trial also showed the association of low serum TGFα with high pCR rates (*p* = 0.04) [47]. Still, in the context of *PI3KCA* mutated status as a predictive factor of pCR, 203 patients from neo-ALTTO trial were screened for different biomarkers of pCR response to anti-HER2 therapy (Lapatinib vs. Trastuzumab vs. Lapatinib+Trastuzumab). Strikingly, presenting only one *PIK3CA* mutation was associated with pCR (OR = 0.42, *p* < 0.0185) in the combined analysis of the three treatment arms [48]. Another study that focused on HER2+ patients (*n* = 254), derived from the neo-ALTTO trial, showed that four different variables were independent predictors of pCR: *ERBB2* gene expression levels, HER2-enriched intrinsic subtype, *ESR1* mRNA levels, and GGI were identified (*p* < 0.001, *p* < 0.001, *p* < 0.008, and *p* < 0.01, respectively) [51].

##### Molecular Signatures as Predictors of Pathological Complete Response

There have been many attempts to identify a molecular signature that could predict pCR. Some of the first studies were from Hess et al. in 2006, who determined the DLDA-30 that predicted pCR after NAC with high sensitivity in a cohort of 133 BC patients (accuracy of 0.76) [57] or from Liedtke et al. in 2009, who identified a 97-gene measure of histological tumor grade, the GGI, which combined the molecular readouts with clinical parameters and predicted pCR with an accuracy of 0.71 [58]. From these studies, a number of initiatives have explored pCR predictors, the most revealing of which are described below (Table 3).

Integrative analysis of copy number and gene expression has also been performed in core biopsies of BC, where gains in 1q (55%), 8q (40%), and 17q (40%) characterize the tumors with pCR after NAC, while 11q11 (37%) was the most abundant gain in tumors from patients that did not achieve pCR. With respect to gene expression variants associated with pCR, *CXCL9*, *AREG*, and *B-MYB* were overexpressed, while ABCG2 was downregulated [59].

Regarding integrative analyses, IntClust classification, based on a combination of transcriptomics and CNAs analysis, was proven useful and more informative than PAM50 for pCR prediction. In a cohort of 871 patients, the IntClust 10 subtype was identified as the one associated with the highest pCR rate (37%), which surpassed the highest rate obtained using PAM50 classification (basal-like, 31%); similarly, the lowest pCR rate detection was performed more accurately with IntClust, as the IntClust 2 subtype obtained a lowest pCR rate of 0%, compared with the lowest pCR rate by PAM50 Luminal A subtype being 6% [60]. This molecular signature was further simplified in a signature, solely based on CNAs, that was able to predict pCR in 7544 breast tumors independent of the histological grade, Ki67 expression or the immunohistochemical subtype (*p* < 0.0015) [47,48,49]. This refined IntClust signature gives rise to 10 different clusters grouped by subtype prognosis. IntClust 1-2-6-9 are luminal poor prognosis, IntClust 3-4-7-8 are luminal good prognosis, IntClust 10 is basal-like, and IntClust 5 is HER2+ [55].

Other innovative approaches to generate good pCR predictors delineate the synergistic contribution of multiple genes to the prediction of pCR. In this sense, in the neo-ALTTO trial, a 41-gene expression signature called the Trastuzumab Risk model (TRAR) signature has been identified as a predictor of response to anti-HER2 therapies in HER2-positive breast cancer patients. TRAR signature score was associated with pCR (AUC = 0.73) when considering three different treatments collectively: Trastuzumab, Lapatinib, and Trastuzumab plus Lapatinib; interestingly, in the scenario of Lapatinib monotherapy, this gene signature increased the pCR prediction potential (AUC = 0.76). Moreover, the combination of TRAR signature and the clinicopathological variables to the prediction algorithm provided greater predictive capability than the one integrating PAM50 signatures and clinicopathological features (AUC = 0.78 vs. 0.74) [56]. Continuing with the HER2+ BC scenario, Carey et al. determined that the high expression of HER2 amplicon genes was associated with pCR in a multivariate analysis in HER2+ tumors with stage II and III under Paclitaxel + Trastuzumab + Lapatinib treatment in a cohort of 305 patients (OR = 1.35, *p* < 0.0252). The p53 mutation signature and IgG signature were also related to pCR (OR = 2.06, *p* < 0.0119 and OR = 1.54, *p* < 0.0024, respectively) [23]. The evaluation of TILs in both cases, IT-TILs and Str-TILs in a HER2+ cohort treated with NAC ± Trastuzumab ± Lapatinib, has been identified as a pCR predictive factor with greater potential than PAM50 subtypes in a study derived from the CherLOB trial (IT-TILs *p* > 0.001 and Str-TILs *p* < 0.001) [25].

In the abovementioned neo-ALTTO trial, of the 714 tested pathways, the mutational status of 33 was associated with response. Relevantly, most of the associated pathways included PI3KCA-related ones, even though the gene was not preferentially mutated, but constituted a central node of a 459 gene network that defined Trastuzumab resistance. Patients with mutations in this network had low pCR rates to Trastuzumab (4%) compared with cases with no mutation (56%), (OR = 0.035; *p* < 0.001). Other pathways where its mutated status was related to pCR included the regulation of RhoA activity. In the Lapatinib arm, mutations in this pathway were related to a high rate of pCR (OR = 14.8, *p* < 0.001) [48]. Another study that focused on HER2+ patients (*n* = 254) derived from the neo-ALTTO trial with 254 patients evidenced the association of *ERRB2* gene expression with pCR. A number of defined gene signatures were sequenced and used for categorizing the patients into three groups based on the expression of *ERBB2* and *ESR1*: high *ERBB2* and low *ESR1*, high *ERBB2* and high *ESR1*, and low *ERBB2*. pCR rate-wise, these groups presented a progressive diminution of 47%, 32%, and 9%, respectively (*p* < 0.001 for the pCR difference between the three groups) [51].

Other molecular signature that uses the best combination of genes to predict pCR constitutes the first epigenetic signature of response to NAC. A whole-genome methylation study in TN breast cancer patients identified the methylation status of nine genes (*LOC641519*, *LEF1*, *HOXA5*, *EVC2*, *TLX3*, *CDKL2*, *FERD3L*, *CHL1*, *TRIP10*) as associated with response in a discovery cohort (*n* = 24). The final signature derived from the analysis in the validation cohort (*n* = 30), was comprised of two genes (*FER3L* and *TRIP10*), where high methylation predicts pCR status (RCB = 0) with an AUC of 0.90 [52].

Finally, the TN molecular classification of Lehmann et al. [61] has been proposed as an independent predictor of pCR [53,54]. In an attempt to validate this finding, we undertook the characterization of a cohort of 125 TN treated with NAC ± Carboplatin in terms of pCR predictors using this TN molecular classification. We identified six molecular subtypes that included Basal-like 1 (BL1), Basal-like 2 (BL2), Immunomodulatory (IM), Mesenchymal (M), Mesenchymal stem-like (MSL) and Luminal androgen receptor (LAR) subtypes. The most proliferative subtype was BL1 (Ki67 > 50% of 88.2% vs. 63.7%, *p* = 0.002), and, consistent with the predictive potential of Ki67, it was the subtype that obtained the highest pCR, particularly when these patients were treated with NAC-Carboplatin (80% vs. 23%, *p* = 0.027). The TN subtype with a lower pCR rate was LAR in all treatments (14.3% vs. 42.7%, *p* < 0.045). Those tumors identified as LAR corresponded with HER2-enriched and luminal A intrinsic subtypes by PAM50, which is consistent with the low pCR rate, since anti-HER2 therapy treatment was not used in this cohort [54]. In 2015, Burstein and collaborators used a thorough molecular approach comprising RNA and DNA profiling analyses to propose a new TN classification with increased prognostic potential. They defined four subtypes that partly overlap with Lehmann subtypes: Basal-like Immunosuppressed Subtype. (BLIS), Androgen receptor (AR), LAR, Mesenchymal subtypes (MES) and Basal-like immune activated subtype (BLIA). Relevant subtype-specific targets are identified, such as MUC1 and AR in LAR subtypes, PDGF in MES subtypes, VTCN1 in BLIS subtype or Stat transduction molecules and cytokines in the BLIA subtype, allowing for stratification based on putative new therapeutic opportunities for the most aggressive BC. Apart from the comparatively improved prognosis value, they define more extensive subtypes that are different in their immunogenic features, BLIS and BLIA [62]. Given that pCR achievement has been correlated with the immunogenic phenotype of the tumor microenvironment, Burstein molecular subtypes should be evaluated for their ability to predict eventual responders to NAC in BC, and possibly outstand PAM50 or Lehmann subtypes as pCR predictors.

## 3. Residual Disease

### 3.1. Residual Disease as a Prognostic Factor in NAC-Treated Breast Cancer Patients

As previously mentioned, the presence of tumor cells after NAC is a fundamental prognostic factor. Several studies revealed that the degree of reduction in tumor burden after NAC is associated with increased DFS and OS [6,7,10,12]. Here, we describe different residual disease biomarkers that are used for the determination of post-NAC and surgery patient outcomes.

#### 3.1.1. Post-Therapy Ki67

In 2009, Jones et al. were pioneers in determining the NAC outcome predictive potential of the proliferation marker Ki67. In a matched cohort of 103 patients, they reached the conclusion that post-chemotherapy Ki67 was a strong outcome predictor in patients without pCR (*p* < 0.001; HR 1.6) [63]. This reduction in Ki67 after treatment with standard NAC has been significantly correlated with better DFS and OS, particularly in the luminal B subgroup of BC. In addition, the multivariate analysis showed that the lack of decrease in Ki67 during NAC significantly increased the hazard ratio for recurrence and death by 3.39 and 7.03, respectively [37].

In GeparTrio, the largest study evaluating the prognostic value of post-NAC Ki67 index in breast cancer, three groups of patients (1151 patients in total; 484 with pCR and 667 with RD) were established based on Ki67: the low Ki67 group (0–15%), the medium Ki67 group (15.1–35%), and the high Ki67 group (>35.1%). The outcome of the low Ki67 group was comparable to the pCR group (*p* < 0.211 for DFS and *p* < 0.779 for OS). The high Ki67 group had a higher risk for disease relapse (*p* < 0.0001) and death (*p* < 0.0001) than the low and medium Ki67 groups [64,65].

#### 3.1.2. Residual Cancer Burden Index

As mentioned before, failure to achieve pCR after NAC is related to worse prognosis [6,9,10,11,12]. For that reason, residual disease has been comprehensively characterized to be used for the stratification of patients according to NAC outcome. The RCB index is a system that quantifies residual disease based on six parameters: two dimensions of the post-treatment breast tumor bed, its cellularity, the percentage of carcinoma in situ, the number of metastatic nodes, and the diameter of the largest nodal metastatic lesion. The application of this index leads to the assignment of four different groups based on the outcome: RCB-0 (pCR), RCB-I (minimal RD), RCB-II (moderate RD), and RCB-III (extensive RD). Symmans et al. (2007) applied this system in a cohort of 382 patients with different treatments and demonstrated that the RBC index was an independent predictor of Distant relapse-free Survival (DRFS), in addition to being reproducible and cost-effective. RCB-0 and RCB-I had similar DRFS (5.4% and 2.4%, respectively), while RCB-III was associated with the longest DRFS (53.6%) [5]. In a recent study, each class of RCB index was thoroughly characterized in a cohort of 399 HER2− patients. In HR+ patients, those with RCB-III were mostly PAM50 luminal A subtype in comparison with patients with RCB-II and RCB-0/I (*p* < 0.01) [66]. Consistent with the fact that ER+ and PR+ BC are less likely to achieve pCR, they observed that the corresponding receptor coding genes (*ESR1* for ER and *PGR* for PR) were overexpressed in RCB-II and RCB-III patients and not in pCR status or RCB-0/I (*p* = 0.00053 and 0.0061, respectively); on the other hand, the expression level of MKI67 was lower in RCB-III than in RCB-0/I and RCB-II (*p* = 0.0029). Finally, in TN subtypes, probably related to the intrinsically unfavorable prognosis of the subtype, RCB-III class was associated with a higher clinical stage and ratio of lymph node-positive than RCB-0/I and RCB-II (*p* < 0.01); also, *ESR1* expression levels were significantly increased in RCB-III tumors with respect to RCB-I and RCB-II tumors (*p* = 0.041) [66].

While RCB can indeed mirror specific PAM50 BC subtypes in terms of response to NAC, other tumor characteristics are independently associated with outcome after NAC and should, therefore, be considered transversally to RCB when characterizing post-NAC tumors. For example, in a cohort of 109 TN patients with RD, multivariate analysis indicated that both CD4+ stromal TILs levels and RCB index have independent prognostic value for Distant recurrence-free interval (DRFI) (CD4+ TIL: HR 2.88, *p* < 0.007 and RCB index: HR 12.04, *p* < 0.0001). High numbers of CD4+ TIL had a benefit on survival across the different classes of RCB RCB-II and RCB-III (*p* < 0.05 for RCB-II, and *p* < 0.05 for RCB-III [67]). However, this is in contrast with a previous study, where TILs only had a positive prognostic effect in RCB-II class tumors, achieving better Relapse-free survival (RFS) (*p* < 0.003) and OS (*p* < 0.008) than RCB-III ones in a cohort of 375 TN patients [68].

#### 3.1.3. Residual Proliferative Cancer Burden

Residual Proliferative Cancer Burden (RPCB) agglutinates the relative events rates for post-therapy Ki67 and RCB. The prognostic value of RPCB has been shown to be much higher than that of either Ki67 or RCB separately, where RPCB efficiently clustered the patients in groups per the RFS and OS rates [64,69]. In a cohort of 220 patients (including both ER+ and ER−), the prognostic information related to time to recurrence of combining Ki67, grade, and ER status to RCB was assessed and quantified as PI. Post-NAC Ki67 was more informative than RCB (χ^2^ = 53.8 vs. χ^2^ = 38.1), although the prognostic information was higher when these variables were taken together under RPCB (χ^2^ = 61.4, *p* < 0.001). Importantly, the prognostic value differs between ER+ and ER− patients. All variables were related to outcome in the ER− subgroup (*p* < 0.001), with the best prognostic variables being RCB (χ^2^ = 35.9) and RPCB (χ^2^ = 35.6). The combination of variables which yielded better prognostic potential was RCB+Ki67+grade+ER status (χ^2^ = 15.3). On the other hand, Ki67 and RCB did not have prognostic potential in the ER+ patients, but only RPCB (χ^2^ = 13.3, *p* < 0.003) [69].

### 3.2. Dynamics of the Molecular Profile of Residual Disease after NAC Treatment and Impact in Outcome

In the post-NAC scenario, it is particularly important to identify the molecular alterations that have been triggered by the treatment and are related to the acquired chemoresistance mechanism. The predominant hypothesis so far is the treatment-induced selection of resistant cancel cell clones, but it is imperative to characterize the tumor molecular dynamics upon treatment to fully determine the NAC-induced resistance beyond the basal predictors of pCR. This would allow the design of more specific treatments with better clinical outcomes [3].

Between 16% and 30% of patients treated with NAC, including those HER2+ that receive NAC-Trastuzumab, undergo changes to biomarker status, such as HR and HER2 status, during treatment, as detected by pre- and post-NAC determinations [3,70].

In addition, these variations are treatment-dependent; for example, 43% of HER2+ patients undergo a loss of HER2 during treatment with NAC-Trastuzumab, versus 9.5% of patients treated only with NAC [3]. The loss of HER2+ status after treatment with anti-HER2 agents could arise from the eradication of HER2+ clones, with the residual disease stemming from pre-existing HER2− populations being attributed high intratumor heterogeneity [3]. Consistently, some studies by Guarneri et al. in 2013 and Mittendorf et al. in 2009 show that the loss of HER2 positivity after NAC correlated with worse DFS (*p* < 0.063) and worse RFS (*p* < 0.041) compared with HER2+ stable patients [3,71,72].

Regarding HR receptors, loss occurs more frequently than gain [70], although gain is associated with a better outcome when compared to the loss or the absence of variation (*p* = 0.039 for DFS; *p* = 0.045 for OS). The multivariate analysis revealed that post-NAC HR status was an independent predictor of DFS (*p* = 0.0078) [73]. In view of this, the post-NAC determination of HR and HER2 status, in patients not expressing them in the pre-NAC primary tumor, should be prioritized [3].

In addition to this prognostic potential of HR dynamics, testing pre- and post-NAC ER, PR, and HER2 is also useful for the identification of actionable variations; indeed, 20% of patients treated with NAC or NAC-Trastuzumab had a clinically actionable change [70].

NAC not only affects the loss or gain of individual receptors, but also induces shifts in PAM50 subtypes. In a cohort of 21 patients (9 Basal-like and 12 non-Basal-like), 62% underwent PAM50 subtype changes after NAC [74]. The most common evolution is towards the normal-like subtype, which could partly be explained by the stromal contamination concomitant to the reduction in tumor cellularity in residual disease [74]. Other studies that are part of clinical trials or that study larger cohorts have reported that the most common transitions in subtypes are luminal B to luminal A [75,76], which is consistent with the preferential elimination of the most proliferative cells by NAC [76]. This hypothesis can find further support in a study where 22.5% of patients evaluated with the gene expression-based assay, Mammaprint, undergo a subtype transition after NAC, primarily from luminal B and HER2 to luminal A. In addition, 76.2% of the transitioned patients switched their Mammaprint risk classification from high risk to low risk, while only 4.8% switched from low risk to high risk and the other 19% did not change [77]. Considering the frequent NAC-induced dynamics of some of the major determinants of pCR, the longitudinal evaluation of such biomarkers during treatment can be considered fundamental for the appropriate management of the neoadjuvant treatment and the ulterior interventions of the residual disease [3].

### 3.3. Genomic and Transcriptomic Profile of Residual Disease

A myriad of studies has evaluated transcriptomic or targeted gene expression patterns in search of predictive markers of clinical outcome after residual disease. Klintman et al. conducted a gene expression study that included the quantification of 24 genes representing key biologic processes related to cancer in 56 pre- and post-NAC paired biopsies, and a total of 126 residual tumors, of which 66% were ER+, 52% PR+, 12% HER2+, 62% ER+/HER2−, and 26% ER−/HER2− [78]. Regarding the post-NAC variations identified in 56 paired biopsies (36 were ER+/HER2− and 13 ER−/HER2−), proliferation genes (MKI67, TOP2A, AURKA, PLAU, STAT1) and the stem cell markers CD44 and STAT3 were significantly downregulated; on the other hand, the EMT genes SNAI1, SNAI2, SOX9, and TWIST; the apoptosis genes PAWR and Bcl2; the Ras–ERK-associated DUSP4 gene; the cell metabolism-related genes ACACB and LDHB; ALDH1A1; the stromal genes DCN and SPARC were all significantly upregulated [78]. This targeted study of the gene expression variant during NAC grouped the tumors with residual disease into four main clusters, which could be representative of the post-NAC scenario. One of them was mainly HR− and HER2−, with high residual Ki67 and MIK67, AURKA, and TOP2A overexpression. The other three clusters were mainly HR+, with low residual Ki67, and generally with the reverse expression pattern; indeed, these tumors were characterized by high levels of ESR1 and genes closely related to ESR1 [78]. In an attempt to determine the inter- and intra-heterogeneity at the epigenetic level, in a recent study (2019), Luo et al. determined that NAC treatment induced alterations of the methylation profile that persisted in the post-NAC scenario. Beyond the inter-patient heterogeneity, four genes related with stem cell quiescence (*ALDH1L1*, *HOPX*, *WNT5A*, and *SOX9*) were hypomethylated after NAC in all patients. This opens a new avenue in the search for alternative post-NAC adjuvant therapeutic approaches [79].

#### Residual Disease Genomic Alterations in Triple Negative Breast Cancer

Only a few studies have addressed the delineation of genomic alterations in the RD of TN patients. For the subtype with highest rates of pCR achievement, when these patients present with RD, the scenario relates more to metastatic recurrence and poorer outcome than the other subtypes that do not tend to achieve pCR. On the other hand, the majority of cells in RD have actionable genetic alterations [80], which is encouraging in the search for new alternatives with targeted therapies and allows for the calculation of more accurate prognosis predictions.

Among the genetic mutations associated with TN RD identified in 2014 by Balko et al. using TCGA data [81], alterations in *TP53* were the most common (89%), followed by *MCL1* (54%) and *MYC* (35%) gene amplifications. *MCL1* amplifications were more frequent in triple negative residual disease than in basal-like primary tumors: 54% in post-NAC vs. 19% in basal-like primary tumors (*p* = 0.0006), indicating that *MCL1* may play a role in de novo or acquired drug resistance [80].

Cell cycle, PI3K/mTOR, GFR, Ras/MAPK, and DNA repair pathways were also altered after NAC (truncations, loss or mutations of *BRCA1*, *BRCA2*, or mutations in *ATM*). Interestingly, the presence of potentially targetable alterations, such as *PTEN* alterations, and *JAK2*, *CDK6*, *CCND1*, *CCND2*, *CCND3*, and *IGF1R* amplifications, indicates that the molecular analysis of TN not achieving pCR to NAC is potentially useful for the identification of therapeutic alternatives [80]. Indeed, the modifications in DNA repair pathways, which included alterations in *BRCA1/2*, are the base for clinical trials such as ECOG-ACRIN EA1131 or CREATE-X [82].

In the analysis of 75 TN RD arising from a phase II trial (BRE09-146), it was observed that the most abundant mutations were also in *TP53* (88%), followed by *PIK3CA* mutations (11%). Regarding the CNA profile of RD, they identified gains in *MCL1*, *MYC*, and *GATA3*, and losses in *BRCA2*, *FLT3* and *RB1*, with the most frequently amplified gene being *MYC* (24% of cases) and the most frequently depleted gene *BRCA2* (68% of cases) [83]. In addition, the analysis of 18 matched pre-NAC vs. post-NAC matched samples showed a mutational homogeneity between both scenarios, given that 13 out of the 18 matched samples had identical mutational profiles. However, this is not the case for the CNA behavior. The NAC modulated the CNA pattern with chaotic acquisition of copy gains and losses, including amplifications in known genes, such as *MCL1*, *MYC*, *KRAS*, *BCL9*, *EGFR*, *CDKN2A*, *BIRC2*, and *BIRC3*, found in the post-NAC scenario compared with the one before treatment [83]. In this study, among the molecular perturbations induced by NAC that predicted recurrence, the loss of *TP53* was associated with a significant DFS and OS decrease (*p* < 0.02 and *p* < 0.03, respectively), highlighting the importance of the loss of locus 17p for TN aggressiveness. Interestingly, *TP53* mutations and MYC signaling upregulation also tend to be concomitant (*p* < 0.05), and related to *MYC* amplification, although only *TP53* mutations correlate with worse DFS and OS (*p* < 0.007 and *p* < 0.007, respectively). Finally, the copy gain of regions in close proximity of *SMAD4* (chromosome 18q) is also associated with poor DFS (*p* < 0.01) [83]. Another comprehensive study by Balko et al. reported the characterization of the gene expression and genetic variants associated with the residual disease phenotype in the TN subtype after NAC using a panel of 450 transcripts that include the signatures of PAM50, MEK and TGFß activation, and post-NAC Ki67 index related genes [80]. The tested expression variants allow the generation of two tumor categories; one includes tumors with higher differentiation, high Ki67, and low TGFß and MEK activity, while the second agglutinates those tumors enriched in genes related to invasive pathways and poor differentiation. Consistent with the stem-like phenotype typically induced by the latter and already related to residual disease [83], this group is characterized by enhanced MEK and TGFß activation, together with the deletion of *DUSP4* [80,84,85,86]. In line with this, additional studies focused on the TN subtype reported the reduction in DUSP4 expression in RD as related to poor RFS (*p* < 0.0004) [84].

Another study evaluating gene expression variants in TN residual disease reports a 7-gene prognostic signature of chemoresistance to NAC [87]. The expression of AR, GATA3, ESR2, GBX2, KRT16, MMP28, and WNT11 in residual disease establishes a risk prediction for a three-year RFS, with 76.9% in the low risk patient category (those with “luminal-like” genes as *AR* or *GATA3*) presenting survival versus 25% of the patients included in the high risk group (those tumors characterized by expression of cancer stem cell markers as KRT16, WNT11, or MMP28). Favorable prognosis was observed in patients presenting high expression of “luminal like” genes (*AR*, *GATA3*) and decreased survival was observed in patients expressing cancer stem cell-like (WNT11) or EMT-associated genes (MMP28) [87]. The status AKT1^low^ of quiescent cancer cells (QCCs) has been related with chemotherapy resistance and progression-inducing epigenetic plasticity in experimental models [88,89]. Kabraji et al. (2017) studied the presence of these cells before NAC, after NAC (RD), and in nodal and distant metastasis in a TN cohort. In patients not responding to NAC, AKT1^low^ QCCs were identified in every one of the tested scenarios, strengthening the status AKT1^low^ as a NAC resistance biomarker [90]. Other reported signatures identified through a multivariable study with TN patients treated with NAC include the expression levels of *ACACB*, *CD3D*, *MKI67*, and *TOP2A* genes, which adds prognostic value to the ER−, PR−, and HER2− status [78]. The identification of TOP2A amplification before NAC in primary tumors as a negative predictor of pCR in a multivariate analysis [49] reinforces the role of *TOP2A* as a chemoresistant gene.

A more ambitious study that analyzed the expression levels of 449 genes related to TN outcome aggressiveness, in a cohort of 82 residual disease TN patients after NAC, defined a 3-gene signature (*CCL5*, *DDIT4*, and *POLR1C*) that was identified as an independent prognostic factor for DRFS in a multivariate analysis. In their cohort, two subgroups (high and low risk) can be discriminated based on the differential DRFS (*p* < 0.002) [91]. CCL5 overexpression was related to good prognosis (*p* < 0.0012), while DDIT4 overexpression was associated with poor prognosis (*p* < 0.00034) in their individual impact evaluation regarding RFS [91].

Finally, as stated previously, the PAM50 expression signature is also the object of modification under NAC. Interestingly, in a series of 21 pre-NAC and post-NAC BC compared by PAM50 subtype using Affymetrix HG-U133A gene chips, the majority of the transitions, departing from any subtype, implied a shift towards normal-like subtype, which may reflect an infiltration of normal tissue within the tumor niche [74].

### 3.4. Towards Preventing and Overcoming NAC Treatment Resistance in Breast Cancer

Due to the high impact pCR achievement has in BC outcome, especially in aggressive subtypes such as TN and HER2, the search for alternatives or combination NAC therapies to prevent residual disease is a priority in the field. As discussed before, in the HER2+ disease, combination with anti-HER2 agents such as Trastuzumab, Pertuzumab, or Lapatinib is known to improve the pCR rate (OR 5.11, *p* < 0.009) [41]. TN disease poses though an increased difficulty in this regard, since identifying a targetable characteristic is a priori challenging. In addition, the important molecular heterogeneity that features this BC subtype represents an additional obstacle [53,61,62]. Notwithstanding that, a few alterations have been identified as putative targets to prevent resistance to NAC in TN cases. The most important molecular alterations are the germline *BRCA 1/2* mutations, or the BRCAness phenotype, that lead to a deficiency in the DNA damage repair machinery, mainly in double strand breaks (DSBs). This deficiency can be utilized in favor of treatment by the addition of platinum salts (Carboplatin) to NAC, since they distort the double helix of DNA inducing single-strand breaks (SSBs) and DSBs, which the tumor cells are not able to repair. This strategy was indeed associated with an improvement in the pCR rate in the CALG 40,603 trial, of 41% vs. 54% (OR 1.71; *p* = 0.0029) [92]. Another cellular mechanism of response to DNA damage that can be targeted to increase the response to NAC is the binding of Poly ADP-ribose polymerase (PARP) to SSBs in the DNA repairing process. The use of PARP inhibitors (PARPi) in BC with BRCA1/2 mutations induces cell death and prevents SSBs repairs. In the I-SPY 2 trial, they evaluated the combination of PARPi plus Carboplatin followed by NAC and only NAC in the other arm of the study in TN breast cancers. As a result, 25% more pCR was achieved with the PARPi and Carboplatin combination (51 vs. 26%), with a concomitant increase in hematologic toxicity [93]. Other approaches that are gaining more relevancy add Immune Checkpoint Inhibition to NAC. ICI based on the inhibition of programmed death-1 (PD-1) receptor or programmed death ligand-1 (PD-L1) aims at restoring the antitumor effects of T cells. TN breast cancer is the BC subtype exhibiting more expression of PD-L1 [72]. In the KEYNOTE-522 trial, the addition of Pembrolizumab (PD-1 inhibitor) induced an increase in pCR rate in TN patients (64.8% vs. 51.2%) [94].

In those cases where NAC is not completely effective, residual disease constitutes a different scenario to study new therapeutic approaches. There are ongoing clinical trials in post-NAC disease trying to identify the best treatment according to the breast cancer subtype. The majority of these studies focus on TN BC due to the lack of targets, although ongoing clinical trials such as PENELOPE-B (NCT01864746) or KATHERINE (NCT01772472) explore new approaches for HR+/HER2− and HER2+ BC, respectively. In the PENELOPE-B trial, the CDK 4/6 inhibitor, Palbociclib, is added to standard endocrine treatment in post-NAC disease in hormone positive BC and in the KATHERINE trial, the combination of Emtansine and Trastuzumab is used as post adjuvant treatment in HER2+ BC. Strategic ongoing clinical trials are the CREATE-X trial, where TN residual disease is treated with Capecitabine and this is translated in better DFS (74.1% vs. 67.6%, HR 0.70, *p* = 0.01) and five-year OS (89.2% vs. 83.6%, HR 0.58, *p* = 0.01) [95]. Other ongoing clinical trials are the A-Brave trial, which evaluates the use of anti-PDL1 in post NAC TN tumors, and the ECOG-ACRIN EA1131 study, where anti-PDL1 is used as adjuvant treatment in Basal-like tumors refractory to platinum salts [82].

## 4. Conclusions

pCR determination in the context of breast cancer neoadjuvant treatment stands out as a useful tool to evaluate new therapeutic approaches. Moreover, pCR prediction has been the focus of a variety of works that aim at identifying decision-making algorithms that can improve the clinical management of breast cancer patients at this stage.

So far, clinical tumor stage, tumor grade, IHC subtypes, or lymphocyte infiltration are the clinicopathological features that are most commonly used to predict response to NAC, due partly to their wide availability in clinical units. However, recent advances in the field of NAC response prediction in breast cancer have unveiled the additional contribution of molecular signatures as pCR predictors, such as PAM50, TRAR signature for HER+ tumors, or IntClust, which exceed the sensitivity of the classical response markers in some cases.

In those cases of incomplete response, the characterization of RD acquires special importance for determining clinical outcome and identifying new actionable targets, given the new tumor scenario induced by NAC. Indeed, it is very relevant that the receptors that are intimately associated with the definition of BC immunohistochemical subtypes (HR and HER2+) and, therefore, the disease prognosis, undergo important rearrangements in their distributions under NAC, and that new tumor drivers arise or are unmasked.

Most of the studies in this respect are devoted to characterizing RD in TN tumors, probably due to their aggressive nature when pCR is not achieved, or because of the lack of specific pharmacological targets. In these tumors, NAC is reported to induce a mostly stochastic CNA rearrangement, together with stem-like phenotypes marked by MEK and TGFβ activation and *DUSP4* deletion.

In view of the high relevance of the pCR predictors for identifying those breast cancer patients who are susceptible to benefiting from NAC, as well as for designing the precise NAC composition, in our opinion, it is of utmost importance to explore new pCR predictors of higher sensitivity and specificity. In addition, expanding the molecular characterization of RD in all BC subtypes will be crucial for understanding the resistance mechanisms to NAC and for the discovery of new actionable targets induced by the treatment.

## 5. Future Perspectives

The incorporation of NAC to the standard of care treatment to early and locally advanced breast cancer has greatly benefited these patients in terms of survival and breast preservation. As for any other novel pharmacological approach, the implementation of NAC has been paralleled by an intense search for those factors influencing its impact on the course of the disease. In an important effort, a myriad of studies has defined different predictors of the most informative prognosis factor, pCR, and characterized the profile of residual disease. Interesting observations include gene expression signatures, some of them subtype-defining, as well as other molecular variations and clinicopathological features that are able to anticipate pCR and, in some cases, prognosis. In addition, a phenotype evolution during treatment has been revealed with a transition towards a “normal-like” phenotype, with losses of the essential breast cancer receptors, or towards the stemness or immune depleted phenotypes as characteristic of those tumors that have not been eradicated by NAC. Despite this significant progress, several concerns are yet to be addressed for an efficient implementation of NAC in terms of predicting the clinical benefit and identifying successive treatments for the residual disease. In both cases, the main pitfall is the important heterogeneity that characterizes this type of cancer; more randomized clinical trials that consider the breast cancer subtypes individually and address the intratumor heterogeneity should evaluate the determinants of pCR and the profile of residual disease. These would generate invaluable input to refine the novel cDNA “mutations tracking” approaches [96,97,98] that require knowledge of the specific predicted residual disease mutations with prognostic potential to be interrogated during the course of NAC and after surgery, and those that could be amenable as targets for ulterior treatments of the residual disease.

Moreover, some pre-clinical initiatives are also emerging to dissect the mechanisms of chemoresistance that can contribute to the identification of additional targets. In this regard, a recent study employed several systems that mimic in vivo TN BC chemoresistance, such as xenograft models, three-dimensional cultures, and primary breast cancer organoids, to identify that Lysyl oxidase (LOX) is a key inducer chemoresistance in TN BC. Indeed, higher LOX was associated with shorter survival in chemotherapy-treated TN breast cancer patients’ organoids [99].

Beyond residual disease, the field is largely lacking deterministic factors of metastasis after NAC and their inter-relation with specific profiles of the primary tumor and residual disease. Indeed, a major concern beyond the detection and prediction of residual disease is the development of distant metastatic disease, which is responsible for 90% of breast cancer-related deaths [100].

To date, a few research studies have established several candidate diagnostic biomarkers of breast cancer metastasis; however, no single predictor of metastasis after residual disease has been identified so far. Therefore, longitudinal studies with homogeneous cohorts controlled for pCR achievement would be key for identification of the impact of NAC in the development of distant metastasis, and the specific tumor evolution towards the most deleterious phenotype of this disease. Considering the current outstanding amount of high throughput generated data related to NAC response, the rational design of the future clinical trials, and the rapid transformation of the real-time non-invasive monitoring technologies, we anticipate the transition in the field towards a more patient- and evolution-specific implementation of NAC for breast cancer.

## Figures and Tables

**Table 1:** Summary of clinical trials based on chemotherapy.

Clinical Trials	Patients	Treatment	pCR (%)
I-SPY [11]	All Subtypes	NAC	HER2-enriched and Basal-like subtypes achieved the best % of pCR compared with Luminal B subtype (55% and 34% vs. 13%, respectively).
GeparDuo [19]	All Subtypes	NAC	Those tumors HR− had better response to NAC than those HR+ (22.8% vs. 6.2% of pCR)
WSG-ADAPT-TN [20]	TN	NAC	In TN, basal-like subtype, High Ki67 and low HER2 score were associated with chemosensitivity (*p* = 0.015, *p* < 0.001 and *p* < 0.001, respectively)
GeparSepto [21]	All Subtypes	NAC	TN breast cancer obtained the best ratio of pCR (48%).
NeoALTTO [22]	HER2+	NAC + (L + T)	51% with dual HER2 therapy versus 30% and 25% with T and L respectively.
CALGB 40,601 [23]	HER2+	NAC + (L + T)	51% with dual HER2 therapy versus 40% and 32% with T and L respectively.
NSABP B-41 [24]	HER2+	NAC + (L + T)	62% with dual HER2 therapy versus 53% and 53% with T and L respectively.
CherLOB [25]	HER2+	NAC + (L + T)	TILs are associated with pCR (OR 1.03; *p* < 0.001). The PAM50 subtype with better pCR ratio was HER2-enriched (50%, *p* < 0.001).
NeoSphere [15]	HER2+	NAC + (*p* + T)	46% with dual HER2 therapy versus 29 and 24 with T and *p* respectively.
TRYPAHENA [26]	HER2+	NAC + (*p* + T)	57–66% with dual HER2 therapy.
BERENICE [27]	HER2+	NAC + T + *p*	The highest pCR rate was in HER2-enriched PAM50 subtype (75%).
NeoPACT	TN	NAC +/− Immune checkpoint inhibitors	Ongoing
GeparNuevo	TN	NAC +/− Immune checkpoint inhibitors	Ongoing
NeoTala	TN	NAC +/− PARP inhibitors	Ongoing
GeparOla	TN	NAC +/− PARP inhibitors	Ongoing

**Table 2:** Studies evaluating PAM50 as a predictive tool for pCR.

Study	Pre-NAC Cohort	Treatment before Surgery	pCR-Achievement Association after Treatment	
Prat et al., 2014 [41]	*n* = 195	NAC-CMF+/−Trastuzumab	HER2-enriched tumors obtain the highest pCR rate when treated with Trastuzumab (OR = 5.11, *p* < 0.009)
	(HER2+ cohort, NOAH trail)		
Prat et al., 2014 [42]	*n* = 1055	NAC	Mostly Basal-like subtype. High expression of proliferation signature and low expression of Luminal A signature show significant association to high rate of pCR (*p* < 0.005 and *p* < 0.023, respectively)
	(TN cohort)		
Prat et al., 2015 [28]	*n* = 957	NAC	In the multivariate analysis, Luminal B, HER2-enriched and Basal-like intrinsic subtypes are related to pCR achievement (*p* < 0.001, *p* < 0.001 and *p* < 0.001, respectively)
	(All subtypes)		
Prat et al., 2016 [43]	*n* = 195	NAC	Luminal A tumors predict low pCR rates compared to the other subtypes (OR 0.341, *p* < 0.037)
	(All subtypes)		
Dieci et al., 2016 [25]	*n* = 121	NAC+/−Trastuzumab+/−Lapatinib	Luminal A subtype presents the lowest pCR rate, while the highest rate is observed in patients with HER2-enriched subtype (*p* < 0.026).
	(HER2+ cohort, CherLOB trial)		
Carey et al., 2016 [23]	*n* = 305	Paclitaxel+Trastuzumab+/−Lapatinib	Luminal A is associated with the lowest ratio of pCR (34%) and HER2-enriched achieves the best rate (70%) (*p* < 0.001)
	(HER2+ cohort, CALGB 40,601 study)		
Llombart-Cussac et al., 2017 [44]	*n* = 151	Trastuzumab + Lapatinib	HER2-enriched tumors correlate with high pCR rates compared to HER2− tumors. (*p* < 0.0004)
	(HER2+ cohort, PAMELA trial)		
Swain et al., 2018 [27]	*n* = 397	NAC+Trastuzumab+Pertuzumab	HER2-enriched tumors achieve the highest pCR rate (around 75%)
	(HER2+ cohort, BERENICE trial)		
Diaz Redondo et al., 2019 [32]	*n* = 259	NAC+Trastuzumab+/−Pertuzumab	Luminal subtypes (A + B) and HER2-enriched are related to high pCR (*p* < 0.008 and *p* < 0.004).
	(HER2+ cohort)		
Ohara et al., 2019 [34]	*n* = 124	NAC	PAM50 signature predicts pCR achievement (univariate *p* < 0.007; multivariate *p* < 0.031)
	(ER+ Cohort)		
Gluz et al., 2020 [20]	*n* = 642	nab-Paclitaxel+Carboplatin or nab-Paclitaxel+Gemcitabine	Basal-like subtype defines patients that more likely achieve pCR than other subtypes (*p* < 0.015)
	(TN cohort, WSG-ADAPT-TN trial)		

**Table 3:** Genomic, transcriptomic, and proteomic predictors of pCR achievement.

Single Gene Variants and Molecular Signature as Predictors of pCR	pCR-Achievement Association after NAC (± antiHER2 Therapy)	Cohort Pre-NAC	Study
SPAG5 transcript and protein	High levels of SPAG5 transcript and protein predict pCR (*p* < 0.024 and *p* < 0.001)	*n* = 508	Abdel-Fatah et al., 2016 [45]
		(All subtypes, MD Anderson-NeoACT)	
		*n* = 200	
		(All subtypes, Nottingham-NeoACT)	
*MYC* amplification	MYC/CEP8 >2.2 predicts pCR AUC = 0.87 (*p* < 0.006)	*n* = 51	Pereira et al., 2017 [46]
		(All Subtypes)	
TGFa protein	Low TGFa protein levels are related with higher pCR rate (*p* < 0.045)	*n* = 107	Bianchini et al., 2017 [47]
		(HER2+ cohort, NeoSphere trial)	
*PIK3CA* mutated state	*PIK3CA* mutated state is associated with reduction in the rate of pCR in HER2+ tumors	*n* = 107	Bianchini et al., 2017 [47]
		(HER2+ cohort, NeoSphere trial)	
	*PI3KCA* mutated state is associated with pCR in the three-condition treatment combined; Lapatinib vs. Trastuzumab vs. Lapatinib+Trastuzumab (OR = 0.42, *p* < 0.0185)	*n* = 203	Shi et al., 2017 [48]
		(HER2+ cohort, neo-ALTTO trial)	
	*PIK3CA* mutated state correlates with lower pCR rates. 38.8% vs. 23% of pCR (*p* < 0.0001)	*n* = 851	Loibl et al., 2019 [49]
		(All subtypes, GeparSepto trial)	
	*PIK3CA* mutated state is associated with low rate of pCR in HER2+ tumors (*p* < 0.006)	*n* = 295	
		(HER2+ cohort, GeparSepto trial)	
*ERBB2* gene	No observation or low level of *ERBB2* amplification is related to less pCR achievement (*p* < 0.048)	*n* = 48	Lesurf et al., 2017 [50]
		(HER2+ cohort, Z1041 trial)	
	High expression of *ERRB2* is predictive of pCR (*p* < 0.001)	*n* = 254	Fumagalli et al., 2017 [51]
		(HER2+ cohort, neo-ALTTO trial)	
	*ERBB2* amplification is associated with high pCR rate (*p* < 0.0001)	*n* = 851	Loibl et al., 2019 [49]
		(All subtypes, GeparSepto trial)	
	*ERBB2* amplification is associated with high pCR rate (*p* < 0.008)	*n* = 295	
		(HER2+ cohort, GeparSepto trial)	
*TOP2A* amplification	*TOP2A* amplification correlates with a reduction in pCR rate (multivariate *p* < 0.036)	*n* = 159	Loibl et al., 2019 [49]
		(TN cohort, GeparSepto trial).	
Two-gene epigenetic score signature	High level of methylation in *FER3L* and *TRIP10* genes predict pCR in TN. (AUC 0.90)	*n* = 54	Pineda et al., 2019 [52]
		(TN cohort)	
TN subtypes	TN subtype is an independent predictor of pCR (*p* < 0.022)	*n* = 146	Masuda et al., 2013 [53]
		(TN cohort)	
	BL1 TN subtype shows the highest rate of pCR to NAC-Carboplatin treatment (*p* < 0.002) while LAR TNBC subtype obtained the lowest rate (*p* < 0.045)	*n* = 125	Santonja et al., 2018 [54]
		(TN cohort)	
IgG signature	IgG immune-cell expression signature is associated with pCR (OR = 1.54, 95% CI 1.16 to 2.05, *p* < 0.0024)	*n* = 265	Carey et al., 2016 [23]
		(HER2+ cohort, CALGB 40,601 trial)	
*p53* mutation signature	*p53* mutation signature is related to pCR (OR = 2.06 95% CI 1.17 to 3.70, *p* < 0.0119)	*n* = 265	Carey et al., 2016 [23]
		(HER2+ cohort, CALGB 40,601 trial)	
HER2 Amplicon Genes	High expression of HER2 amplicon genes is associated with pCR in HER2+ Tumors (OR = 1.35, 95% CI 1.04 to 1.77, *p* < 0.0252)	*n* = 265	Carey et al., 2016 [23]
		(HER2+ Cohort, CALGB 40,601 Trial)	
TIL	TIL detection in the tumor stroma and intratumor is a predictive factor of pCR (*p* < 0.001 and *p* < 0.001, respectively)	*n* = 121	Dieci et al. 2016 [25]
		(HER2+ cohort, CherLOB trial)	
PIK3CA network genes	Patients with a mutation in the PIK3CA network genes are less likely to achieve pCR in the Trastuzumab arm (4% vs. 56%), OR = 0.035; *p* < 0.001	*n* = 203	Shi et al., 2017 [48]
		(HER2+ cohort, neo-ALTTO trial)	
*ERBB2/ESR1* signature	The combination of high expression of *ERBB2* and low expression of *ESR1* defines a group of patients with high rate of pCR (*p* < 0.001)	*n* = 254	Fumagalli et al., 2017 [51]
		(HER2+ cohort, neo-ALTTO trial)	
Regulation of RhoA activity pathway	Mutation in RhoA pathway is related to a high rate of pCR to Lapatinib treatment (OR = 14.8, *p* < 0.001)	*n* = 203	Shi et al., 2017 [48]
		(HER2+ cohort, neo-ALTTO trial)	
10-IntClust Classification	IntClust subtypes are associated with pCR (multivariate *p* < 0.0015)	*n* = 100	Alba et al., 2018 [55]
		(All subtypes, GEICAM/2006-03, GEICAM/2006-14)	
TRAR signature score	Expression levels of a 41-gene signature predict pCR (AUC = 0.73)	*n* = 226	Di Cosimo et al., 2019 [56]
		(HER2+ cohort, neo-ALTTO trial)	

## Abbreviations

**Abbreviation**	**Meaning**
ABCG2	ATP-binding cassette transporter G2
ACACB	Acetyl-CoA carboxylase beta
Akt	Protein kinase B
ALDH1A1	Aldehyde dehydrogenase 1 family member A1
AR	Androgen receptor
AREG	Amphiregulin
ATM	Ataxia telangiectasia mutated
ATP	Adenosine triphosphate
AUC	Area under the curve
AURKA	Aurora kinase A
B-MYB	Myb-related protein B
BC	Breast cancer
BCL2	B-cell lymphoma 2
BCL9	B-cell CLL/lymphoma 9
BIRC2	Baculoviral IAP repeat containing 2
BIRC3	Baculoviral IAP repeat containing 3
BL1	Basal-like 1
BL2	Basal-like 2
BLIA	Basal-like immune activated subtype
BLIS	Basal-like immunosuppressed subtype
BRCA1	Breast cancer gene 1
BRCA2	Breast cancer gene 2
CCL5	Chr. chemokine ligand 5
CCND1	Cyclin D1
CCND2	Cyclin D2
CCND3	Cyclin D3
CD20	Cluster of differentiation 20
CD3	Cluster of differentiation 3
CD37	Cluster of differentiation 37
CD3D	Cluster of differentiation 3 delta subunit
CD4	Cluster of differentiation 4
CD44	Cluster of differentiation 44
CDK4	Cyclin-dependent kinase 4
CDK6	Cyclin-dependent kinase 6
CDKN2A	Cyclin-dependent kinase inhibitor 2A
CEP	cTerminally encoded peptide
CMF	Cyclophosphamide/Methotrexate/Fluorouracil
CNAs	Copy number alterations
cT-stage	Clinical tumor stage
CXCL9	Chemokine (C-X-C motif) ligand 9
CXCR3	C-X-C motif chemokine receptor 3
DCN	DECORIN
DDIT4	DNA-damage-inducible transcript 4
DFS	Disease-free survival
DLDA-30	30 probe diagonal linear discriminant analysis
DRFI	Distant recurrence-free interval
DRFS	Distant relapse-free survival
DSBs	Double strand breaks
DUSP4	Dual specificity phosphatase 4
EFS	Event-free survival
EGFR	Epidermal growth factor receptor
EMT	Epithelial-to-mesenchymal transition
ER	Estrogen receptor
ERBB2	Erb-B2 Receptor Tyrosine Kinase 2
ERK	Extracellular signal-regulated kinase
ESR1	Estrogen receptor alpha
ESR2	Estrogen receptor 2
FLT3	Fms-related receptor tyrosine kinase 3
FOXO3a	Forkhead box O3
GATA3	GATA binding protein 3
GBX2	Gastrulation brain homeobox 2
GFR	Growth factor receptor
GGI	Genomic grade index
HER2	Human epidermal growth factor receptor type 2
HOPX	HOP homeobox
HR	Hormone receptor
ICI	Immune checkpoint inhibition
IGF1R	Insulin-like growth factor 1 receptor
IHC	Immunohistochemistry
IM	Immunomodulatory
IntClust	Integrative cluster
IT-TIL	Intratumor-tumor infiltrating lymphocytes
JAK2	Janus kinase 2
Ki67	Antigen Ki67
KRAS	Kirsten rat sarcoma viral oncogene homolog
KRT16	Keratin 16
LABC	Locally advanced breast cancer
LAR	Luminal androgen receptor
LDHB	Lactate dehydrogenase B
LOX	Lysyl oxidase
M	Mesenchymal
MAPK	Mitogen-activated protein kinase
MCL1	Myeloid cell leukemia 1
MEK	Mitogen-activated protein kinase kinase
MES	Mesenchymal subtype
MKi67	Marker of proliferation Ki-67
MMP28	Matrix metallopeptidase 28
MSL	Mesenchymal stem-like
mTOR	Mechanistic target of rapamycin kinase
MUC1	Mucin 1, cell surface associated
MYC	Master regulator of cell cycle entry and proliferative metabolism
NAC	Neoadjuvant chemotherapy
OR	Odds ratio
OS	Overall survival
PAM50	Prediction analysis of microarray 50 subtypes
PARP	Poly ADP-ribose polymerase
PARPi	PARP inhibitor
PAWR	Pro-apoptotic WT1 regulator
pCR	Pathological complete response
PD-1	Programmed D-1
PD-L1	Programmed D ligase-1
PDGF	Platelet-derived growth factor
PI	Prognostic index
PI3K	Phosphatidylinositol 3-kinase
PI3KCA	Phosphatidylinositol 3-kinase catalytic subunit alpha
PLAU	Plasminogen activator, urokinase
POLR1C	RNA polymerase I And III Subunit C
PR	Progesterone receptor
PTEN	Phosphatase and tensin homolog
QCCs	Quiescent cancer cells
RB1	Retinoblastoma protein 1
RCB	Residual cancer burden
RD	Residual disease
RFS	Relapse-free survival
RhoA	Ras homolog family member A
RPCB	Residual proliferative cancer burden
SMAD4	SMAD family member 4
SNAI1	Snail family transcriptional repressor 1
SNAI2	Snail family transcriptional repressor 2
SOX9	SRY-box transcription factor 9
SPAG5	Sperm-associated antigen 5
SPARC	Secreted protein acidic and cysteine-rich
SSBs	Single strand breaks
STAT	Signal transducer and activator of transcription
STAT1	Signal transducer and activator of transcription 1
STAT3	Signal transducer and activator of transcription 3
Str-TIL	Stromal-tumor infiltrating lymphocytes
TCGA	The Cancer Genome Atlas
TGFa	Transforming growth factor alpha
TGFb	Transforming growth factor beta
TILs	Tumor infiltrating lymphocytes
TN	Triple negative
TOP2A	Topoisomerase IIA
TP53	Tumor protein 53
TRAR	Trastuzumab risk model signature
TWIST	Twist basic helix-loop-helix transcription factor
VTCN1	V-set domain containing T cell activation inhibitor 1
WNT11	Wnt family member 11
WNT5A	Wnt family member 5A
